# Reduced NK Cell Cytotoxicity by Papillomatosis-Derived TGF-β Contributing to Low-Risk HPV Persistence in JORRP Patients

**DOI:** 10.3389/fimmu.2022.849493

**Published:** 2022-03-08

**Authors:** Xunyao Wu, Yang Xiao, Dan Guo, Zixin Zhang, Meiyu Liu

**Affiliations:** ^1^ Clinical Biobank, Department of Medical Research Center, Peking Union Medical College Hospital, Chinese Academy of Medical Sciences and Peking Union Medical College, Beijing, China; ^2^ Department of Otolaryngology Head and Neck Surgery, Beijing Tongren Hospital, Capital Medical University, Beijing, China

**Keywords:** NK cells, JORRP, TGF-β1, HPV6, HPV11

## Abstract

The role of natural killer (NK) cells in juvenile-onset recurrent respiratory papillomatosis (JORRP) patients remains elusive. In this study, we find increased NK cell percentage, particularly CD11b-CD27- (DN) subsets in peripheral blood of JORRP patients and associated with disease activity. RNA sequencing shows a downregulated “natural killer cell-mediated cytotoxicity” feature in JORRP tumors. We also find impaired cytotoxic capacity and lower expression of NK cell-activating receptors including NKp30 and NKp46. Higher transforming growth factor-beta 1 (TGF-β1) is found both in plasma and tumor tissues of JORRP, and anti-TGF-β1 antibody could restore NK cell cytolytic activity and upregulate NKp30 and NKG2D expression. Also, we find a significantly higher Chemokine receptor type 6 (CXCR6) on NK cells in tumors compared with that in peripheral blood. Finally, RT-PCR analysis show that both HPV6-E6-E7 and HPV11-E6-E7 overexpression leads to higher *TGFB1* expression compared with control SNU-1076 cell line, and higher CXCR6 expression is detected on NK coculture with HPV11-E6-E7-overexpressing cells. In conclusion, we demonstrate that TGF-β1 by papillomatosis leads to decreased NK cell cytotoxicity through downregulating NK cell-activating receptors in JORRP patients.

## Introduction

Human papillomavirus (HPV) is a double-stranded oncogenic DNA virus that mainly infects keratinocytes and mucosal epithelium and consists of more than 200 types (1). HPV carries two oncogenes, E6 and E7, which contribute to tumor growth and carcinogenesis (2). In children, HPV accounts for a number of distinct diseases including cutaneous warts, genital warts, and squamous intraepithelial lesions (3). Juvenile-onset recurrent respiratory papillomatosis (JORRP), primarily caused by low-risk HPV6 and HPV11 infection, is considered as the most common benign laryngeal tumor in children that manifests with hoarseness and loss of voice (4). Moreover, repeated costly surgical interventions place a lifetime disease burden on families (5, 6).

A number of studies show that impaired/dysregulated immune responses account for the failure of HPV clearance and severity of RRP disease. One study shows defective gene expressions related to Th1 response in RRP patients (7). A recent published work finds that monocyte and Langerhans cell innate immunity is impaired in RRP patients (8). The study of cytokine mRNA profiles in peripheral blood mononuclear cells suggests dysregulated cytokine mRNA response and impaired cytotoxic capacity of JORRP patients (9).

Natural killer (NK) cells are well-known innate members involved in the surveillance and elimination of virus infection and cancers (10). NK cells are featured with natural cytotoxicity and heterogeneous populations that could be divided into interferon (IFN)-γ-producing CD56^bright^ and cytotoxic CD56^dim^ populations (11). NK cell cytotoxicity against transformed cells is majorly triggered by a number of activating receptors including NKG2D and the Natural Cytotoxicity Receptor (NCR) family that is composed of NKp30, NKp46, and NKp44 (12). Flow cytometry analysis of NK cells suggests that downregulated expressions of NK cell-activating receptors NKp30, NKp46, and NKG2D contribute to HPV evasion in high-risk HPV-16-associated cervical cancer and squamous intraepithelial lesion patients (13).

To date, limited information is available on the role of NK cells in the pathogenesis of JORRP patients. Our present study shows that enhanced expression of TGF-β contributes to downregulated expression of activating NK receptors including NKp30, thus decreasing the cytotoxicity of NK cells against tumors in JORRP patients.

## Materials and Methods

### Patient Recruitment and Cell Lines

Peripheral blood and tissue samples were obtained from JORRP patients under surgical interventions at Beijing Tongren Hospital from September 2015 to July 2021. Peripheral blood samples of age- and sex-matched healthy donors were obtained from children under physical examination for admission to kindergarten or elementary school at Beijing Tongren Hospital from September 2015 to July 2021. The criterion of disease activity assessment was performed according to what has been previously described (14). The demographic characteristics of clinical information are summarized in **Supplementary Table S1**.

K562 (RRID: CVCL_0004) was purchased from ATCC, and SNU-1076 (RRID: CVCL_5006) was purchased from Korean Cell Line Bank (Seoul, South Korea). Both human cell lines have been authenticated using short tandem repeat profiling within the last 3 years. All experiments were performed with mycoplasma-free cells.

### Human Papillomavirus Genotyping

Total DNA was extracted from papillomas, and HPV genotyping was performed by real-time PCR as previously described. The cycle threshold (Ct) was calculated, and in the present study, Ct ≤30 was considered HPV positive (15).

### Peripheral Blood Mononuclear Cell and Tumor-Infiltrating Cell (Tumor-Infiltrating Lymphocyte) Isolation

Peripheral blood mononuclear cells (PBMCs) were isolated by centrifugation through Ficoll gradients as previously described (16). Tumor tissue samples were cut into small pieces and digested in RPMI 1640 (HyClone, USA) supplemented with collagenase IV (3 mg/ml, Sigma-Aldrich, USA) and DNase I (0.1 mg/ml, Sigma-Aldrich, USA) at 37°C for 1 h, passed through 70-μm cell strainer, centrifuged, and then resuspended in phosphate-buffered saline (PBS) medium for further flow cytometry analysis.

### Flow Cytometry

The cells were incubated with Fc blockade reagent for 20 min at room temperature and stained with fluorochrome-conjugated antibodies in serum and sodium azide-containing buffer. Flow cytometry was performed as previously described (16). Antibodies in the present study were listed as follows: FITC anti-CD56 (Cat#362546, BioLegend, USA), PE anti-NKp30 (Cat#325208, BioLegend, USA), anti-CD56 (Cat#362508, BioLegend, USA), anti-NKp44 (Cat#325108, BioLegend, USA), anti-CD27 (Cat#356406, BioLegend, USA), PerCP-Cy5.5 anti-CD3 (Cat#317336, BioLegend, USA), anti-NKG2D (Cat#320818, BioLegend, USA), APC anti-NKp46 (Cat#331918, BioLegend, USA), anti-CD11b (Cat#301310, BioLegend, USA), anti-CXCR6 (Cat#356006, BioLegend, USA), anti-CD3 (Cat#317318, BioLegend, USA), PE-Cy7 anti-NKp46 (Cat#331916, BioLegend, USA), anti-CD3 (Cat#317334, BioLegend, USA), APC-Cy7 anti-CD3 (Cat#317342, BioLegend, USA), and anti-CD45 (Cat#304014, BioLegend, USA). FlowJo software was used for data analyzing (Tree Star, Inc., Ashland).

### Analysis of RNA Sequencing Data

Fresh-frozen tissue samples were obtained from JORRP patients under surgical intervention. Total RNA was extracted with TRIzol reagent following the manufacturer’s instructions (Invitrogen). RNA sequencing of JORRP tumors and paired adjacent nontumor tissues (n = 4) was conducted by GENEWIZ (Suzhou, China) at the Illumina NovaSeq 6000 platform with 150 length as previously reported (17). Raw reads that contained sequencing adapters, those with a quality score less than 20, those shorter than 50, or artificial reads were filtered.

### Differentially Expressed Genes and Enrichment Analysis

Packages DESeq2 (V1.6.3) and edgeR implemented in BioConductor were used to analyze the differentially expressed genes (DEGs) in tissues and cell lines in the R environment, respectively (11). DEGs were determined by absolute (log2 fold change) >1 and an adjusted P value <0.05. We utilized the package “clusterprofiler” to conduct Gene Set Enrichment Analysis (GSEA) and visualized the results with the package “ggplot 2” in the R environment (12).

### Flow Cytometry-Based Measurement of Natural Killer Cell Degranulation

Viable PBMCs were counted, and NK cell percentage was determined by flow cytometry. For some experiments, NK cells were purified by immunomagnetic negative selection (NK cell isolation kit, Miltenyi Biotec) according to manufacturers’ instructions. Then, numbers of PBMCs/purified NK cells and K562 cell lines were adjusted to ensure the ratio of NK:K562 = 1:1. Mixed cells were resuspended in RPMI 1640 medium with 100 U/ml penicillin and 100 μg/ml streptomycin supplemented with 10% fetal bovine serum (FBS; Gibco, NY, USA). PE anti-human CD107a (Cat#328608, BioLegend, USA) or APC anti-human CD107a (Cat#328620, BioLegend, USA) antibody with 5 μl/test was directly added to the coculture medium and incubated at 5% CO_2_ and 37°C for an additional 4 h. Then, samples were washed and stained with anti-CD3 and anti-CD56 antibody for 20 min at room temperature. After washing two times, the expression of surface CD107a was analyzed by flow cytometry.

### Cytometric Bead Assay and ELISA

Cytokines [interleukin (IL)-1α, IL-2, IL-10, IL-6, IL-12p70, IL-15, IL-33, interferon (IFN-γ)] in plasma were determined by cytometric bead assay (CBA) inflammation kit (BD Biosciences) and TGF-β1 by ELISA (Cusabio) according to manufacturers’ instructions as previously reported (18).

### Immunohistochemistry

The conventional streptavidin-peroxidase method was performed according to the manufacturer’s protocols (Dako Denmark, K8002, Beijing, China). The primary antibody anti-TGF-β1 (Abcam, ab215715, 1:200 dilution) was used for immunohistochemistry (IHC). Stained sections were visualized and captured with a ×20 objective using an Axio Scan.Z1 microscope (Zeiss, Germany).

### Construction of HPV6-E6-E7- or HPV11-E6-E7-Overexpressing Cell Lines

SNU-1076 was maintained in Dulbecco’s modified Eagle’s medium (DMEM) with 100 U/ml penicillin and 100 μg/ml streptomycin supplemented with 10% FBS (Gibco, NY, USA) at 5% CO_2_ and 37°C. The construction of HPV6-E6-E7- and HPV11-E6-E7-overexpressing cell lines were conducted as previously described (17).

### Natural Killer/SNU-1076 Cell Coculture System

Purified NK cells were sorted by magnetic sorting using NK cell isolation kit (130-092-657, Miltenyi Biotec). Here, 1× 10^4^/200 μl human NK cells were seeded in 96-well plates in the presence of 100 U/ml IL-2 (PeproTech) with either 50 nM LY364947 (Abmole BioScience) or control dimethylsulfoxide (DMSO) for 3 days, then stimulated with 1× 10^4^ K562 cells for another 6 h. For SNU-1076 coculture system, 1 ml 2 × 10^5^ human PBMCs plus 1 × 10^5^ indicated SNU-1076 cell lines were seeded in 24-well plate for 6 days in the presence of 100 U/ml IL-2. Then, cells were harvested for subsequent flow cytometry analysis.

### RNA Isolation and Real-Time PCR

Total RNA was extracted from indicated cell lines using the Qiagen RNeasy Kit (74034) following the manufacturer’s instructions. Real-time PCR was performed using TB Green Premix Ex Taq™ (Takara, Japan) in an ABI-7500 Real Time PCR system (Applied Biosystems, CA, USA). A detailed description of the primers used for TGF-β1 is listed as follows: TGF-β1-F: CCCAGCATCTGCAAAGCTC; TGF-β1-R: GTCAATGTACAGCTGCCGCA; GAPDH-F: ATCAAGAAGGTGGTGAAGCA; GAPDH-R: GTCGCTGTTGAAGTCAGAGGA.

### Statistical Analysis

GraphPad Prism 8.0 software (La Jolla, CA) was used for statistical analysis. All data were presented as the mean ± SD. Comparison of differences between two independent groups was analyzed by Student’s *t* test. The unpaired Student’s *t* test was used if values followed a normal distribution and the Mann–Whiney test otherwise. Paired *t* test was used to analysis differences in **Figures 5B–E**. Comparison of differences among multiple groups was analyzed by one-way ANOVA. The Tukey’s test was used if values followed a normal distribution, and the Dunnett’s test was used otherwise. Correlations were evaluated by the nonparametric Spearman rank correlation test. A P value <0.05 was considered statistically significant.

## Results

### Increased CD56^dim^ Natural Killer Cells in the Peripheral Blood and Are Associated With Disease Activity in Juvenile-Onset Recurrent Respiratory Papillomatosis Patients

**Figure 1:**
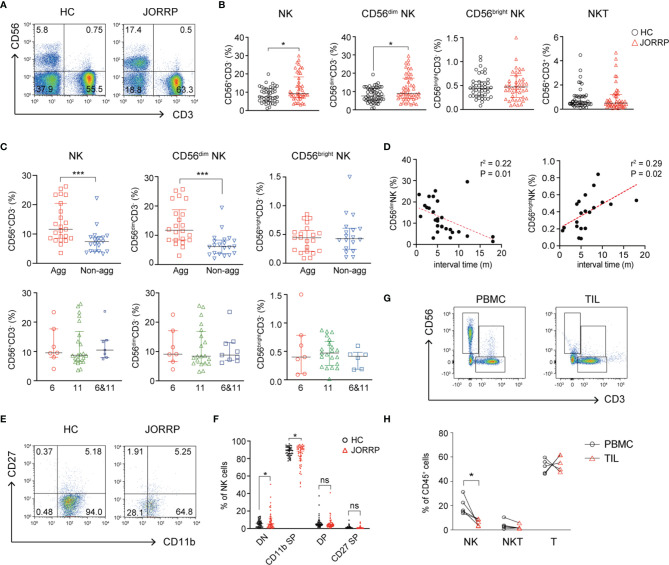
CD56^dim^ NK cells are cumulated in the peripheral blood and correlated with disease activity in Juvenile-Onset Recurrent Respiratory Papillomatosis (JORRP) patients. **(A, B)** Representative graphs and percentage analyses of NK cells in the peripheral blood of JORRP patients (n = 48) and HCs (n = 51). **(C)** Percentage analysis of NK cells in the PBMCs of different subgroups of JORRP patients. Agg, aggressive (n = 23); Non-agg, non-aggressive (n = 19); 6, HPV6 (n = 7); 11, HPV11 (n = 22); 6&11, HPV6&HPV11 (n = 7). Note: Patients with recurrence were not included in the aggressive/non-aggressive group. The genotyping of some JORRP patients was undefined due to limited tumor volume. **(D)** Correlation of CD56^dim^ NK or CD56^bright^ NK cell percentages with interval time of surgical reoccurrence. Spearman’s correlation coefficients are shown. m, months. **(E, F)** Representative graphs and flow cytometry analyses of the expression of CD27 and CD11b on gated CD56^+^CD3^-^ NK cells in the peripheral blood of JORRP patients (n = 48) and HCs (n = 48). DN, CD27 and CD11b double-negative NK cells; CD11b SP, CD11b single positive NK cells; DP, CD27 and CD11b double-positive NK cells; CD27 SP, CD27 single-positive NK cells. **(G, H)** Representative graphs and percentage analyses of NK cells in the peripheral blood and tumor of JORRP patients (n = 5). PBMC, peripheral blood mononuclear cell; TIL, tumor-infiltrating lymphocyte. Data are presented as median with interquartile range. Each dot represents a single patient. ns, not significant; *P < 0.05; ***P < 0.001.

We first analyze the percentage of NK cells in the PBMCs from healthy controls (HCs) and JORRP patients. As shown in **Figures 1A, B**, the percentages of total CD56^+^CD3^-^ NK cells and CD56^dim^ NK cells in the peripheral blood of JORRP patients are significantly higher than that in the HCs. We further compare the percentages of total NK, CD56^dim^ NK, and CD56^bright^ NK in different subgroups of JORRP patients based on disease severity or HPV genotyping. Of note, the percentages of total CD56^+^CD3^-^ NK cells and CD56^dim^ NK cells are higher in JORRP patients of aggressive disease activity but not differing from those of different HPV genotypes (**Figure 1C**). Linear regression analysis shows that CD56^dim^ NK cell percentage is negatively correlated and CD56^bright^ NK cell percentage is positively correlated with interval time of reoccurrence (**Figure 1D**). We utilize CD11b and CD27 to define distinct stages of NK cells as described in a previous study (19). Interestingly, we find that nearly all the NK cells from peripheral blood of HCs display CD11b^+^CD27^-^ phenotype (CD11b^+^ SP), however, we find increased percentage of CD11b-CD27- (DN) NK cell populations present in the peripheral blood of JORRP patients compared with HCs (**Figures 1E, F** **)**. In contrast to the augmented percentage of NK cells in the peripheral blood, the percentage of NK cells in tumor-infiltrating lymphocytes (TILs) is significantly lower compared with PBMCs in the same JORRP patient (**Figures 1G, H** **)**.

### Lower Natural Killer Cell Cytolytic Activity in Juvenile-Onset Recurrent Respiratory Papillomatosis Patients

**Figure 2:**
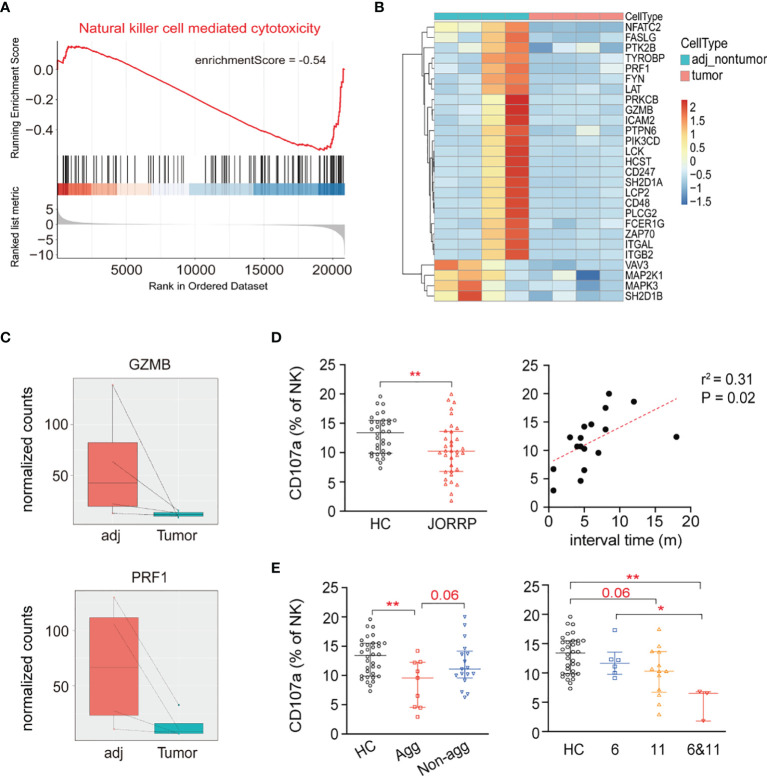
Decreased NK cell cytotoxicity in JORRP patients. **(A)** The GSEA plot of natural killer cell-mediated cytotoxicity pathway downregulated in tumors of JORRP tissues. **(B)** The heatmap showing specific expression of natural killer cell-mediated cytotoxicity pathway genes in JORRP tissues and paired adjacent nontumor tissues (n = 4). **(C)** Bar plots showing the normalized counts of *GZMB* and *PRF1* genes in adjacent nontumor tissues (adj) and tumor tissues. **(D)** Percentage analysis CD107a expression on NK cells determined by flow cytometry of HCs (n = 34) and JORRP patients (n = 34). Correlation of CD107a expression on NK cells with interval time of surgical reoccurrence. Spearman’s correlation coefficients are shown. m, months. **(E)** Percentage analysis of CD107a expression on NK cells determined by flow cytometry of HCs and different subgroups of JORRP patients. HCs, healthy controls (n = 34); Agg, aggressive (n = 9); Non-agg, non-aggressive (n = 17); 6, HPV6 (n = 6); 11, HPV11 (n = 13); 6&11, HPV6&HPV11 (n = 3). Data are presented as median with interquartile range. Each dot represents a single patient. ns, not significant; *P < 0.05; **P < 0.01.

We perform GSEA of RNA sequencing data between the JORRP tumors (n = 4) and paired adjacent normal tissues and find downregulated “natural killer cell-mediated cytotoxicity” signaling pathway in JORRP tumors (**Figure 2A**, enrichmentScore = -0.54). We next focus our attention on the negatively enriched “natural killer cell-mediated cytotoxicity” signaling pathway in JORRP tissues. The expression levels (including log2 fold change and adjusted P value) of 26 genes related to the signaling pathway are shown in **Figure 2B** and **Supplementary Table S2**. Of note, the expressions of *GZMB* and *PRF1*, which are essential effector molecules for NK cell cytotoxicity, are found significantly lower in tumor tissues compared with adjacent nontumor tissues of JORRP patients (**Figure 2C**).

To validate whether NK cell-mediated cytotoxicity is lower in JORRP patients, we isolate PBMCs from JORRP and HCs, coincubate with K562 cells for an additional 4 h, and measure the surface expression of CD107a gated on CD56^+^ NK cells by flow cytometry. We find that the CD107a expression of the NK cells present in PBMCs of JORRP patients is significantly lower than that of HCs and positively correlated with interval time of reoccurrence (**Figure 2D**). Also, the CD107a expression of NK cells is lower in PBMCs from JORRP patients with aggressive disease activity or with HPV6 and HPV11 co-infection (**Figure 2E**). Based on the above results, we conclude that the cytotoxicity of NK cells is lower in JORRP patients.

### Decreased Expression of Natural Killer Cell-Activating Receptors (NKp30 and NKp46) in Juvenile-Onset Recurrent Respiratory Papillomatosis Patients

**Figure 3:**
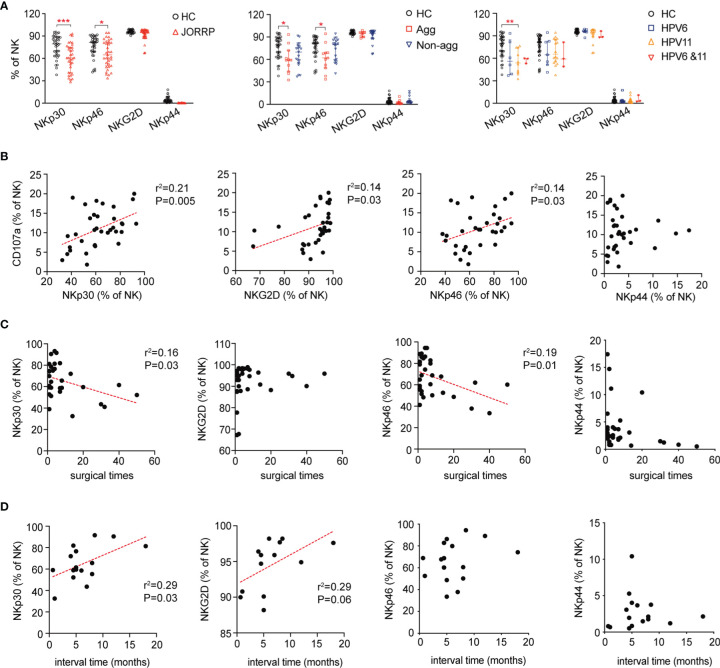
Decreased expression of NK cell-activating receptors on NK cells in JORRP patients. **(A)** Percentage analysis of NKp30, NKp46, NKG2D, and NKp44 expression on NK cells as determined by flow cytometry of HCs (n = 35) and JORRP patients (n = 35) or different subgroups of JORRP patients. Agg, aggressive (n = 11); Non-agg, non-aggressive (n = 19); 6, HPV6 (n = 14); 11, HPV11 (n = 16); 6&11, HPV6&HPV11 (n = 3). **(B)** Correlation of CD107a expression on NK cells with NKp30, NKp46, NKG2D, and NKp44 expression on NK cells. Spearman’s correlation coefficients are shown. **(C)** Correlation of NKp30, NKp46, NKG2D, and NKp44 expression on NK cells with surgical times. Spearman’s correlation coefficients are shown. **(D)** Correlation of NKp30, NKp46, NKG2D, and NKp44 expression on NK cells with interval time of surgical reoccurrence. Spearman’s correlation coefficients are shown. Data are presented as median with interquartile range. Each dot represents a single patient. ns, not significant; *P < 0.05; **P < 0.01; ***P < 0.001.

We next analyze the expression of NK cell-activating receptors including NKp30, NKG2D, NKp46, and NKp44 by flow cytometry. In agreement with impaired NK cell cytotoxicity, the expression of NK cell-activating receptors including NKp30 and NKp46 is lower in NK cells of JORRP patients (**Figure 3A**). Furthermore, we find that NKp30 expression is lower in JORRP with aggressive disease or with HPV11 infection (**Figure 3A**). NKp46 expression is lower in JORRP with aggressive disease (**Figure 3A**). Correlation analysis shows that the CD107a expression on NK cells is positively correlated with NKp30, NKG2D, and NKp46 expression (**Figure 3B**). NKp30 or NKp46 expression shows a significantly negative correlation with surgical times (**Figure 3C**). NKp30 or NKG2D expression shows a significantly positive correlation with interval time of reoccurrence (**Figure 3D**). Collectively, these observations indicate that downregulated NK cell-activating receptors, particularly of NKp30, on NK cells in JORRP patients may affect their ability to eliminate virus-infected tumor cells.

### Papillomatosis-Derived TGF-β Is Involved in Inhibiting Natural Killer Cell Cytotoxicity

**Figure 4:**
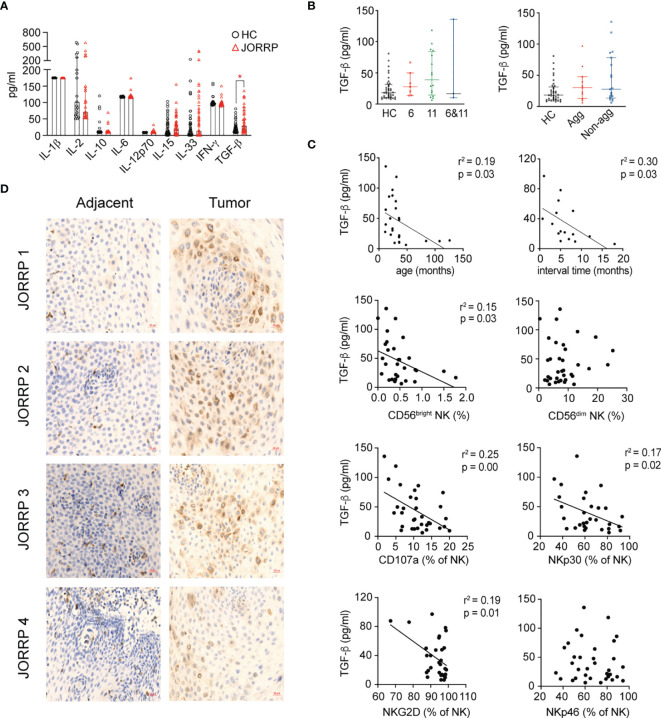
Higher Transforming Growth Factor (TGF)-β level in plasma and papillomatosis of JORRP patients and associated with decreased NK cell cytotoxicity. **(A)** Measurement of multiple cytokine concentrations in plasma of HCs and JORRP patients by CBA (IL-1α, IL-2, IL-10, IL-6, IL-12p70, IL-15, IL-33, IFN-γ) and TGF-β1 by ELISA. **(B)** Immunohistochemistry of TGF-β1 in tumor sections (right) and paired adjacent nontumor sections (n = 4). Bar: 20 μm. **(C)** Concentration analysis of TGF-β1 in different subgroups of JORRP patients. Agg, aggressive (n = 11); Non-agg, non-aggressive (n = 19); 6, HPV6 (n = 7); 11, HPV11 (n = 16); 6&11, HPV6&HPV11 (n = 3). **(D)** Correlation of plasma TGF-β1 with age of first occurrence, interval time, the percentage of CD56^bright^ and CD56^dim^ NK, CD107a, NKp30, NKp46, NKG2D, and NKp44 expression on NK cells. Spearman’s correlation coefficients are shown. Data are presented as mean ± SD. Each dot represents a single patient. *P < 0.05.

Cytokines in the tumor microenvironment are pivotal for the activation, “repression/exhaustion,” and function of NK cells (20). To explore the role of cytokines in NK cell function of JORRP patients, we perform cytometric bead assay (CBA) detection of plasma cytokines including IL-1β, IL-2, IL-10, IL-6, IL-12p70, IL-15, IL-33, IFN-α, and TGF-β that might be involved in modulating NK cell function. We find significantly higher concentrations of TGF-β in plasma of JORRP patients than HCs (**Figure 4A**). A similar level of TGF-β is detected in plasma of JORRP patients with different disease activity or with different HPV genotype. (**Figure 4B**). Moreover, inverse linear relationships are observed between plasma TGF-β concentration and the age of first occurrence, interval time, percentage of CD56^bright^ NK, NKp30, or NKG2D expression on circulating NK cells of JORRP patients (**Figure 4C**). In particular, the papillomatosis epithelium exhibits a stronger TGF-β−positive signal than adjacent nontumor tissue detected by IHC (**Figure 4D**).

**Figure 5:**
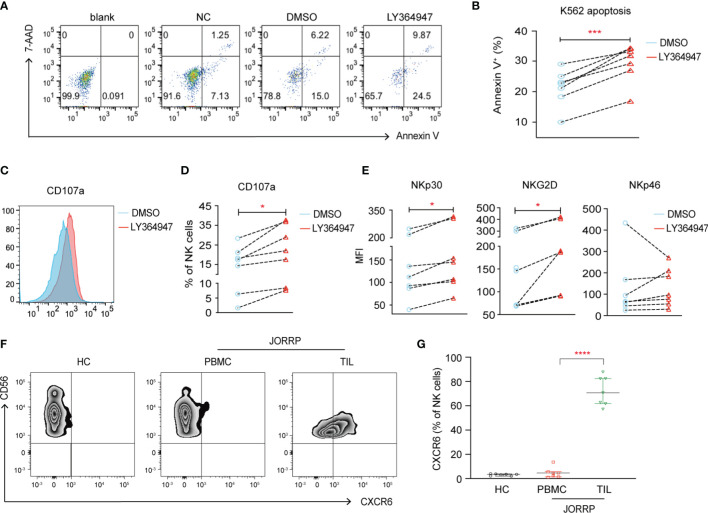
TGF-β1 mediates decreased NK cell cytotoxicity. **(A)** NK cells purified from PBMCs of JORRP patients were preincubated with LY364947 for 3 days in the presence of 100 U/ml IL-2, then stimulated with K562 target cells for an additional 6 h at the ratio = 1:1. **(A, B)** Representative paragraph and percentage analysis of K562 cell apoptosis (the percentage of Annexin V on K562 cells) was determined by flow cytometry. Blank, K562 cells alone; NC, NK cells only; DMSO, NK cells + DMSO; LY364947, NK cells + LY364947. **(C, D)** Representative histograms and percentage analysis of CD107a on NK cells. **(E)** NKp30, NKG2D, and NKp46 expression on NK cells. MFI, mean fluorescent intensity. **(F, G)** PBMCs from HCs, PBMCs and TILs were isolated from JORRP patients. Representative paragraph and percentage analysis of CXCR6 gated on CD56^+^CD3^-^ cells. TIL, tumor-infiltrating lymphocyte. Data are presented as median with interquartile range. Each dot represents a single individual. *P < 0.05; ***P < 0.001; ****P < 0.0001.

To further investigate whether higher TGF-β in plasma or papillomatosis accounts for impaired NK cell cytotoxicity in JORRP patients, we culture purified NK cells from peripheral blood of JORRP patients either alone or in the presence of TGF-βR1 inhibitor LY364947. The cytolytic activity of NK cells is partially restored in the coculture experiments with increased K562 apoptosis (Annexin V^+^) and surface CD107a expression on NK cells when TGF-βR1 activity is blocked by TGF-βR1 inhibitor LY364947 (**Figures 5A–D**). In agreement with data above, we find TGF-βR1 inhibitor LY364947 treatment results in enhanced surface expressions of NKp30 and NKG2D (**Figure 5E**).

Notably, we find a significantly higher expression of CXCR6 on CD3^-^CD56^+^ cells among the TILs than the PBMCs of the same JORRP patient; however, the expression of CXCR6 on CD3^-^CD56^+^ cell in the PBMCs is similar between JORRP patients and HCs (**Figures 5F, G** **)**.

### The HPV6 and HPV11 Oncogenes E6 and E7 Affect Natural Killer Cell Accumulation and Expression of Natural Killer Cell-Activating Receptors

**Figure 6:**
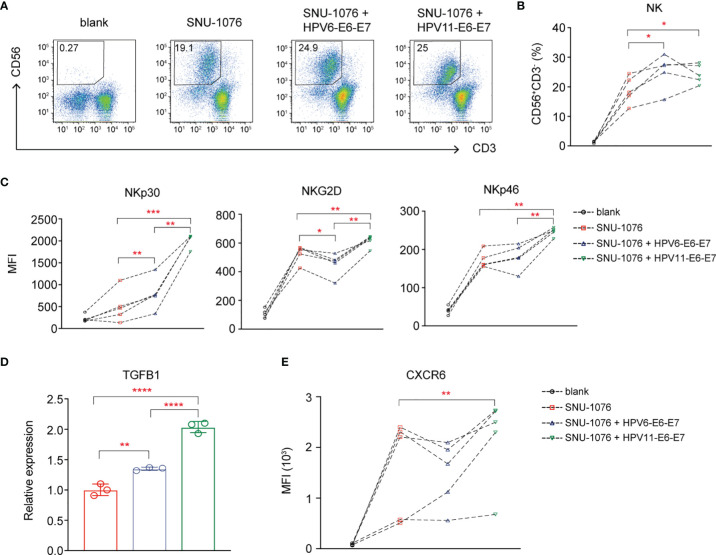
The Human papillomavirus (HPV)6 and HPV11 E6 and E7 oncogenes affect NK cell cumulation and NK cell-activating receptor expression. PBMCs of HCs were cultured for 6 days with 100 U/ml IL-2 either in the absence (blank) or in the presence of the indicated SNU-1076 HNSCC cell lines. **(A, B)** Representative plots and percentage analysis of CD56^+^CD3^-^ NK cells. **(C, E)** NKp30, NKG2D, and NKp46 expression on NK cells. **(D)** Relative expression of *TGFB1* by RT-PCR. MFI, mean fluorescent intensity. Blank, PBMCs alone; SNU-1076, PBMCs cocultured with SNU-1076 cell line; SNU-1076 + HPV6-E6-E7, PBMCs cocultured with HPV6-E6-E7-overexpressing SNU-1076 cell line; SNU-1076 + HPV11-E6-E7, PBMCs cocultured with HPV11-E6-E7-overexpressing SNU-1076 cell line. Each dot represents a single individual. *P < 0.05; **P < 0.01; ***P < 0.001; ****P < 0.0001.

Finally, the effects of E6 and E7 oncogene of HPV6 and HPV11 on the NK cell function are explored. We coculture PBMCs from HCs with stably overexpressed HPV6-E6-E7 and HPV11-E6-E7 or control SNU-1076 HNSCC cell line. Flow cytometry analysis of NK cell percentage and activating receptors shows that both HPV6-E6-E7 and HPV11-E6-E7 gene overexpression results in increased NK cell percentage and NKp30 expression (**Figures 6A–C**). In particular, HPV11-E6-E7 overexpression but not HPV6-E6-E7 gene overexpression leads to increased expression of NKG2D and NKp46 on NK cells (**Figure 6C**). RT-PCR analysis shows that both HPV6-E6-E7 and HPV11-E6-E7 overexpression display higher *TGFB1* expression, with greater expression in HPV11-E6-E7-overexpressing cell lines (**Figure 6D**). Finally, we detect a higher expression of CXCR6 on NKs cocultured with HPV11-E6-E7-expressing cells lines (**Figure 6E**). Taken together, our data suggest a role for the influence of HPV6 or HPV11 E6 and E7 oncogenes in inducing accumulation and activation on NK cells and TGF-β1 expression in JORRP patients.

## Discussion

In our present study, we demonstrate decreased NK cell cytotoxicity with downregulated NK cell-activating receptors in JORRP patients. Our results also indicate that HPV6 or HPV11 virus infection induces papillomatosis secretion of TGF-β1 that subsequent reduces NK cell cytotoxicity.

Currently, the role of NK cells in the HPV-associated disease is still not well elucidated. A previous study finds that NK cells infiltrate in HPV-associated preneoplastic cervical lesions and display higher cytotoxic activity and cytokine production against HPV-like particles (VLPs) (21). Another study reports that HPV16 virus could disable the NK cell increase in early lesions of the cervix (22). Downregulated NKp30, NKp46, and NKG2D expression and reduced cytotoxic activity on NK cells are found in cervical cancer (13). To our knowledge, we are the first to systematically study the percentage, phenotype, and function of NK cells in peripheral blood and tumor tissue of HPV6 and HPV11 virus-associated papillomatosis. Here, we show increased percentage of NK cells in peripheral blood and significantly decreased percentage of NK cells in paired tumor tissues of JORRP patients. These observations indicate that low-risk HPV might prevent NK cell chemotaxis and decrease NK cell cytotoxic ability to facilitate immune evasion in tumors of JORRP patients.

While nearly all of the NK cells from HCs display CD11b^+^ SP mature phenotype, we detect increased frequency of CD11b^-^CD27^-^ (DN) immature NK cell populations in peripheral blood of JORRP patients. DN NK cells are reported to display a more immature phenotype with highly expressed NKG2A and lower NKG2C, NKG2D, CD11c, CD7, and CD2 than the other three NK subsets. Function analysis shows that DN NKs produced the lowest levels of IFN-γ and TNF-α as well as cytolytic ability (19). A previous study detects a substantial proportion of DN NK subsets, and they exhibit a poor cytotoxic capacity and deficient ability to produce IFN-γ in tumor tissue from patients with hepatocellular carcinoma (HCC) and associate with tumor progression (23). Evidence from previous studies suggests that the impairment of NK cell maturation is associated with low-risk HPV persistence and reoccurrence of JORRP. However, since CD16, CD57, and KIR expressions, which reflected the maturation of NK cells, are not detected and increased percentage of CD11b^-^CD27^-^ NK cells is indeed insufficient to draw the conclusion of an immature population of NK cells, further studies with more phenotyping markers are still needed to explore whether JORRP patients are indeed with NK cell developmental defect or persistent low-risk HPV infection prevents the maturation of NK cells. In a previous study by Marcoe et al. (24), mice whose NK cells lack TGF-β receptor (TGF-βR) signaling have more fully mature NK cells, indicating that TGF-β is responsible for NK cell immaturity during infancy. Therefore, it is an interesting topic to further investigate whether higher TGF-β level during infancy prevent NK cell maturation in JORRP patients. Also, TGF-β has been found to induce exhaustion of effector memory T cells in B-cell non-Hodgkin’s lymphoma and NK cells in liver cancer (11, 25); therefore, it is also interesting to confirm whether low-risk HPV infection could induce tumorigenic cells to produce TGF-β or other factors that increase exhaustion of cytotoxic cells in our future study.

TGF-β and related signaling pathway factors are previously found associated with HPV-related diseases. Iancu et al. (26) observe that cervical lesions without HPV infection express significantly less TGF-β1. The E6 protein of HPV5 and HPV8 is found to be able to inhibit TGF-β and NOTCH signaling. Inhibition of TGF-β and NOTCH signaling is linked to delayed differentiation and sustained proliferation of differentiating keratinocytes (27). Chen et al. (28) demonstrate that high-risk HPV E7 could bind to the TGF-β promoter region, resulting in TGF-β overexpression and subsequent Smad4 signaling pathway activation. Our study is the first to show a higher TGF-β level in plasma and tissue of JORRP patients and the potential ability of HPV11-E6-E7 protein in inducing *TGFB1* expression.

Various literature reports support the role of TGF-β in downregulating NK cell-activating receptors, especially NKG2D-mediated cytolytic ability (18, 29). In our study, we observe that plasma TGF-β concentration is negatively correlated with NKp30 or NKG2D expression on NK cells from JORRP patients, and blockade of TGF-βR1 results in enhanced surface expression of NKp30 and NKG2D by *in vitro* study. The comprehensive immunosuppressive microenvironment of tumors affected NK cell-mediated killing. Not only the cytokines that measured in **Figure 4A** (IL-1β, IL-2, IL-10, IL-6, IL-12p70, IL-15, IL-33, IFN-γ, and TGF-β) could affect NK cell cytotoxicity, other soluble factors including indoleamine 2,3-dioxygenase (IDO), prostaglandin E2 (PGE2), lactate, and adenosine could also negatively regulate maturation, proliferation, and effector function of NK cells (30). Moreover, the direct interplay between inhibitory receptors (including immune checkpoint receptors) and the ligands on cancer cells could also inhibit NK cell cytotoxicity (31). Therefore, although we observe increased TGF-β in JORRP patients that conversely linked to reduced NK cell receptor expression and NK cell function that can be partially restored by TGF-β blockade, there might be other potential soluble factors that synergized with TGF-β in different subgroups of JORRP patients.

Besides inhibiting NK cell activity and function, recent studies also demonstrate a role of TGF-β in driving conversion of NK cells into ILC1-like cells to blunt tumor surveillance (32, 33). We demonstrate a higher CXCR6 expression of NK cells in tumor of JORRP patients and enhanced CXCR6 expression on NK cells cocultured with HPV11-E6-E7-overexpressing SNU-1076 cell lines. Moreover, the mRNA level of *TGFB1* is significantly higher in HPV11-E6-E7-overexpressing SNU-1076 cell lines. Also, Gerein et al. (34) found that RRP patients with HPV11 genotyping displayed a more aggressive disease course and a lower incidence of long-term response to IFN-alpha therapy (14% of HPV11 vs. 64% of HPV6). However, a recent study by Flommersfeld et al. (35) provided evidence that ILC1-like NK cells are a transcriptionally, phenotypically, and functionally distinct NK cell lineage, but they seem to not derive from NK cytotoxic cells, but from an independent lineage (36); therefore, further studies are still needed to provide evidence that the E6 and E7 oncogenes of HPV11 virus facilitate tumor immunoevasion through TGF−β1-induced conversion of NK cells.

In summary, our present studies demonstrate that low-risk infection might induce secretion of TGF−β1 by papillomatosis that leads to reduced NK cell cytotoxicity through downregulating NK cell-activating receptors and consequently facilitates low-risk HPV11 persistent infection in JORRP patients. GC1008 (fresolimumab), a human anti-TGF−β monoclonal antibody, has been tested in patients with advanced malignant melanoma, renal cell carcinoma, and diabetic nephropathy and demonstrated acceptable safety and preliminary evidence of antitumor activity (37, 38). Our present study provides evidence for the role of TGF-β1-mediated immunoevasion in JORRP patients and potential therapeutic strategies for clinic JORRP therapy.

## Data Availability Statement

The original contributions presented in the study are publicly available. This data can be found here: https://ngdc.cncb.ac.cn/gsa-human/browse/HRA001665.

## Ethics Statement

This study has been conducted according to Declaration of Helsinki principles. The study was approved by the Ethics Committee of Beijing Tongren Hospital (No. TRECKY2020-157). Written informed consent was obtained from the parents/legal guardians of all the patients.

## Author Contributions

XW designed the study. XW, ZZ, and ML performed the experiments. XW analyzed the data. XW, YX, and DG gave technical and material support. XW and YX drafted the article. All authors read and approved the final article. The work reported in the paper has been performed by the authors, unless clearly specified in the text.

## Funding

This work was supported by the National Natural Science Foundation of China (81601439).

## Conflict of Interest

The authors declare that the research was conducted in the absence of any commercial or financial relationships that could be construed as a potential conflict of interest.

## Publisher’s Note

All claims expressed in this article are solely those of the authors and do not necessarily represent those of their affiliated organizations, or those of the publisher, the editors and the reviewers. Any product that may be evaluated in this article, or claim that may be made by its manufacturer, is not guaranteed or endorsed by the publisher.
