# Supplementary material for: Reduced NK Cell Cytotoxicity by Papillomatosis-Derived TGF-β Contributing to Low-Risk HPV Persistence in JORRP Patients
Source: Front Immunol. 2022 Mar 8;13:849493. doi: 10.3389/fimmu.2022.849493 (PMC8957810; doi:10.3389/fimmu.2022.849493)
Supplement: Supplementary file 1 [file Table_1.docx]

**Supplementary Table I. Demographic and clinical characteristics of JORRP patients**

| Variables | Parameters |
| --- | --- |
| Gender (Male/Female) | 32/27 |
| Age (Year) | 7.9 ± 5.4 |
| HPV genotyping | |
| HPV6 | 11 (18.6%) |
| HPV11 | 35 (59.3%) |
| HPV6 & HPV11 | 13 (22.1%) |
| Surgery interventions | |
| N < 10 | 37 (62.7%) |
| N >= 10 | 22 (37.3%) |
| Age at onset | |
| <= 3 year | 45 (76.3%) |
| > 3 year | 14 (23.7%) |
