# Supplementary material for: Reduced NK Cell Cytotoxicity by Papillomatosis-Derived TGF-β Contributing to Low-Risk HPV Persistence in JORRP Patients
Source: Front Immunol. 2022 Mar 8;13:849493. doi: 10.3389/fimmu.2022.849493 (PMC8957810; doi:10.3389/fimmu.2022.849493)
Supplement: Supplementary file 2 [file Table_2.docx]

**Supplementary Table II. Natural killer cell mediated cytotoxicity signaling pathway-related genes**

| Gene Name | Log2FoldChange | adjust p-value |
| --- | --- | --- |
| *PTK2B* | -0.74 | 0.135 |
| *MAP2K1* | -0.76 | 0.101 |
| *MAPK3* | -0.92 | 0.066 |
| *FCER1G* | -0.94 | 0.117 |
| *PTPN6* | -1.14 | 0.077 |
| *TYROBP* | -1.47 | 0.030 |
| *LAT* | -1.49 | 0.036 |
| *PRF1* | -1.64 | 0.036 |
| *SH2D1B* | -1.67 | 0.025 |
| *FASLG* | -1.79 | 0.048 |
| *GZMB* | -1.88 | 0.012 |
| *ICAM2* | -1.90 | 0.027 |
| *PIK3CD* | -1.90 | 0.018 |
| *LCP2* | -1.97 | 0.011 |
| *FYN* | -2.35 | 0.002 |
| *NFATC2* | -2.88 | 1×10^-13^ |
| *ITGB2* | -2.95 | 0.0009 |
| *PLCG2* | -3.08 | 0.001 |
| *CD247* | -3.35 | 0.0002 |
| *HCST* | -3.39 | 0.0004 |
| *LCK* | -3.44 | 0.0004 |
| *SH2D1A* | -3.49 | 0.002 |
| *ZAP70* | -3.51 | 8.8×10^-5^ |
| *ITGAL* | -3.81 | 9.3×10^-5^ |
| *CD48* | -4.60 | 6.3×10^-6^ |
| *PRKCB* | -4.65 | 3.6×10^-5^ |
